# A fashion product recommendation based on adaptive VPKNN-NET algorithm without fuzzy similar image

**DOI:** 10.3389/fdata.2025.1557779

**Published:** 2025-08-07

**Authors:** R. Sabitha, D. Sundar

**Affiliations:** ^1^Department of Computer Science, Madurai Kamaraj University, Madurai, Tamil Nadu, India; ^2^P.G. Department of Computer Science, Government Arts and Science College, Madurai, Tamil Nadu, India

**Keywords:** recommendation model, collaborative filtering, K-Nearest Neighbor, Principal Component Analysis, convolution neural network, e-commerce

## Abstract

**Introduction:**

Recommender systems are essential in e-commerce for assisting users in navigating large product catalogs, particularly in visually driven domains like fashion. Traditional keyword-based systems often struggle to capture subjective style preferences.

**Methods:**

This study proposes a novel fashion recommendation framework using an Adaptive VPKNN-net algorithm. The model integrates deep visual feature extraction using a pre-trained VGG16 Convolutional Neural Network (CNN), dimensionality reduction through Principal Component Analysis (PCA), and a modified K-Nearest Neighbors (KNN) algorithm that combines Euclidean and cosine similarity metrics to enhance visual similarity assessment.

**Results:**

Experiments were conducted using the “Fashion Product Images (Small)” dataset from Kaggle. The proposed system achieved high accuracy (98.69%) and demonstrated lower RMSE (0.8213) and MAE (0.6045) compared to baseline models such as Random Forest, SVM, and standard KNN.

**Discussion:**

The proposed Adaptive VPKNN-net framework significantly improves the precision, interpretability, and efficiency of visual fashion recommendations. It eliminates the limitations of fuzzy similarity models and offers a scalable solution for visually oriented e-commerce platforms, particularly in cold-start scenarios and low-data conditions.

## 1 Introduction

The global expansion of e-commerce has transformed how businesses and consumers interact, enabling the digital buying and selling of goods and services ranging from apparel to financial products (Rosário and Raimundo, 2021; Erisman, 2017). Platforms such as Amazon, Alibaba, and Flipkart have leveraged data, automation, and global logistics to streamline retail operations and improve customer experience (Ukaj et al., 2020). For small and medium enterprises, e-commerce reduces entry barriers and expands market reach without the need for physical storefronts (Taher, 2021).

E-commerce platforms offer numerous advantages. These include 24/7 accessibility, broader product variety, reduced infrastructure costs, international reach, and targeted advertising opportunities (Kumar et al., 2000; Wu and Gereffi, 2018; Al Tamer, 2021). Businesses can utilize data analytics to deliver personalized recommendations and re-engage customers through intelligent retargeting strategies (Le and Liaw, 2017). For consumers, e-commerce offers unparalleled convenience and the ability to compare products across a wide spectrum.

Despite these strengths, e-commerce also introduces significant challenges. Users cannot physically inspect products prior to purchase, leading to potential dissatisfaction (Sharma and Lijuan, 2015). Delays in delivery, limited real-time customer support, over-reliance on stable technology infrastructure, and increased exposure to cybersecurity threats are persistent concerns (Costa and Castro, 2021; Aseri, 2021; Rosaci and Sarné, 2012). Additionally, the e-commerce landscape is highly competitive due to low barriers to entry, requiring businesses to innovate continuously to retain customer attention (Alyari and Jafari Navimipour, 2018).

These shifts have fundamentally impacted consumer behavior, driving demand for intelligent, personalized digital experiences (Tintarev and Masthoff, 2010). Within this context, Recommender Systems (RS) play a critical role by guiding users to relevant products based on behavioral and contextual data. RSs are widely employed across e-commerce, media streaming, and social platforms to increase user engagement, satisfaction, and sales (Zhou and Zou, 2023; Shin and Darpy, 2020).

However, traditional RS models, which rely on keyword matching or collaborative filtering, often fail in visually driven domains like fashion. These methods struggle to capture subjective and stylistic preferences embedded in product images. As a result, recent research emphasizes visually aware recommender systems that can interpret product aesthetics more accurately (Jiang and Benbasat, 2004). Emerging models that leverage deep learning for personalized fashion recommendations (Vattikonda et al., 2025) highlight the growing demand for visually-aware systems. Anwar et al. (2022) addressed cold-start and sparsity challenges using collaborative filtering integrated with kNN strategies.

This study addresses the limitations of fuzzy logic-based image similarity and explores a deep learning-based solution. We propose a novel Adaptive VPKNN-net algorithm, which combines a pre-trained VGG16 Convolutional Neural Network (CNN) for deep feature extraction, Principal Component Analysis (PCA) for dimensionality reduction, and a dual-distance K-Nearest Neighbors (KNN) model utilizing both Euclidean and Cosine similarity metrics.

This approach overcomes the limitations of textual features and enables efficient visual recommendation, even in small datasets. Our contributions are summarized as follows:

1. Development of the Adaptive VPKNN-net algorithm that integrates CNNs with refined similarity metrics.
2. Elimination of fuzzy image similarity methods in favor of precise, interpretable visual similarity assessment.
3. Demonstration of model effectiveness using the “Fashion Product Images Small” dataset, highlighting strong performance with limited training data (Sengupta et al., 2020).

By improving the precision and interpretability of fashion recommendations, this system enhances the user experience and offers actionable insights for online retailers.

## 2 Related works

Zhang et al. (2019) presented an early deep learning survey on recommender systems, which was later expanded in a multimodal framework for fashion recommendation by Zhang et al. (2025). Recent studies, such as Mathews et al. (2022), have demonstrated the effectiveness of deep feature extraction combined with similarity matching for image-based retrieval tasks (Table 1).

**Table 1:** Comparative summary of recent research works in fashion product recommendation.

**References**	**Proposed method/goal**	**Key technologies/models used**	**Evaluation datasets**	**Limitations/future work**
Zhang et al. (2025)	Recommendation of fashion outfit combinations (tops and bottoms) by integrating price features with image and textual information using a multimodal deep learning model.	Multimodal Deep Learning Model (DeepFMP), Price Feature Extraction Module, Multi-head Attention Mechanism, Visual Features, Text Features	Real user outfit data; Various evaluation metrics (demonstrated improvements), Top-10 recommendations (improved price alignment)	Future work includes more diverse datasets (fabric, material, color, style, age, region, culture, social influences), refining clothing categories (beyond tops/bottoms), and using advanced pre-trained models (Vit, Llama, CLIP).
Florea et al. (2025)	To explore how visual merchandising and store layout influence customer shopping decisions, identify key) shaping consumer behavior, and provide insights for retailers using interactive marketing factors.	Conceptual model combining visual merchandising and store layout factors, analyzed using SmartPLS for Structural Equation Modeling (SEM).	Survey data from 488 respondents in the South Muntenia region of Romania; Statistical analysis (e.g., β coefficients) to determine influence of factors like product layout (β = 0.570), exterior lighting (β = 0.517), window displays (β = 0.510), names and signs (β=0.470), and mannequins (β=0.430).	Sample size limited to 488 respondents from South Muntenia (limits generalizability); Reliance on self-reported data (potential for bias). Future research directions include: examining larger, more diverse samples; using eye-tracking technology or observational techniques; exploring impact of AR/VR on visual merchandising; investigating cultural differences in customer responses to store elements.
Alamdari et al. (2022)	Developed a recommendation algorithm based on images to recommend items.	VGG16, Xception, ResNet50, Inception V3 (CNNs for latent feature extraction), Cosine Similarity, Item KNN	Four different MovieLens datasets (unspecified versions); Accuracy, Mean Absolute Error (MAE), Root Mean Square Error (RMSE)	The lack of discussion on cold-start problems for new items or users is also a potential limitation.
Walek and Fajmon, 2023	Developed a hybrid e-shop recommendation system combining a content-based model with an expert system and three subsystems.	Content-based model, Expert system, Collaborative filtering (subsystem 1), Purchase history analysis (subsystem 2)	N/A (focus on system design)	Reliance on purchase history posed challenges for new users and sparse data; could limit discovery of novel products.
Feldman et al. (2022)	Analyzed the effectiveness of two approaches for identifying ideal products for display on Alibaba platforms (Tmall, Taobao).	Company's existing practice (ML algorithm without customer substitution), Logit framework (MNL)	Historical data from MNL framework; Revenue (MNL outperformed ML); Heterogeneous-effect analyses (MNL performed particularly well for single-purchase customers)	MNL model's superior performance for single-purchase customers suggests potential limitations in addressing repeat buyers with evolving preferences.
Nadkarni and Dasararaju, 2022	Presented a comprehensive visual search solution for reseller commerce.	Novel triplet mining technique (considering relative data order), Triplet semi-hard method, Multi-task learning	N/A (focus on attribute retrieval and extraction)	Overlooked user experience; specific production environment constraints not explicitly detailed; lacked details on specific algorithms used for comparison with individual learning methods.
Iwendi et al. (2022)	Aimed to create a machine-learning model that assigned product scores based on the number of reviews and comments received.	Machine learning model (unspecified), Similarity algorithm	Yelp data (11 metropolitan areas); Mean Accuracy (79%), Precision (79%)	Did not specify the “standard algorithm” used for comparison; focusing solely on review/comment count might overlook sentiment or quality.

## 3 Enhancing visual fashion recommendation with adaptive VPKNN-net

This research introduces a novel fashion product recommendation system that leverages the “Fashion Product Images Dataset Small” from Kaggle. At its core, the system utilizes a pre-trained Convolutional Neural Network (CNN), specifically VGG16, to extract relevant and rich visual features from product images. Recognizing that these deep features often present high dimensionality, Principal Component Analysis (PCA) is then employed to reduce their dimensionality while crucially preserving the most significant information, thereby enhancing computational efficiency. For the recommendation stage, the K-Nearest Neighbors (KNN) algorithm is utilized. This algorithm, which functions as a modified K-Nearest Neighbors algorithm within the broader framework (though the specific “VPKNN-NET” adaptive component is not explicitly detailed here, it implies a refined KNN approach for visual product similarity), calculates image similarity by leveraging these reduced feature vectors. It does so by considering both cosine and Euclidean distance metrics, allowing for a comprehensive assessment of visual likeness. Ultimately, the system retrieves and presents the ‘k' closest images based on these calculated distances as recommendations. This study thoroughly investigates the effectiveness of this combined approach, integrating CNN-based feature extraction, PCA-based dimensionality reduction, and KNN-based similarity calculation, to construct a robust and efficient fashion product recommendation system.

This research proposes a novel two-stage approach for fashion product recommendations, termed the Adaptive VPKNN-net Algorithm. The first stage leverages a pre-trained VGG16 Convolutional Neural Network (CNN) for robust feature extraction from product images. This utilizes transfer learning, where the VGG16, originally trained on a vast dataset like ImageNet, is repurposed to generate high-dimensional feature vectors that capture the intricate visual characteristics of fashion items. Subsequently, Principal Component Analysis (PCA) is employed for dimensionality reduction, efficiently compressing these high-dimensional feature vectors while preserving their most vital information. The second stage uses these reduced feature vectors within a modified K-Nearest Neighbors (KNN) framework for recommendation. This Adaptive VPKNN-net approach dynamically calculates visual similarity using both Euclidean and Cosine distance metrics. This allows the system to identify the ‘k' closest items to a query, providing precise and interpretable recommendations by moving beyond ambiguous fuzzy similarity methods, even with limited training data. The overall aim is to develop a highly accurate and efficient system for recommending visually similar fashion products.

**Figure 1:**
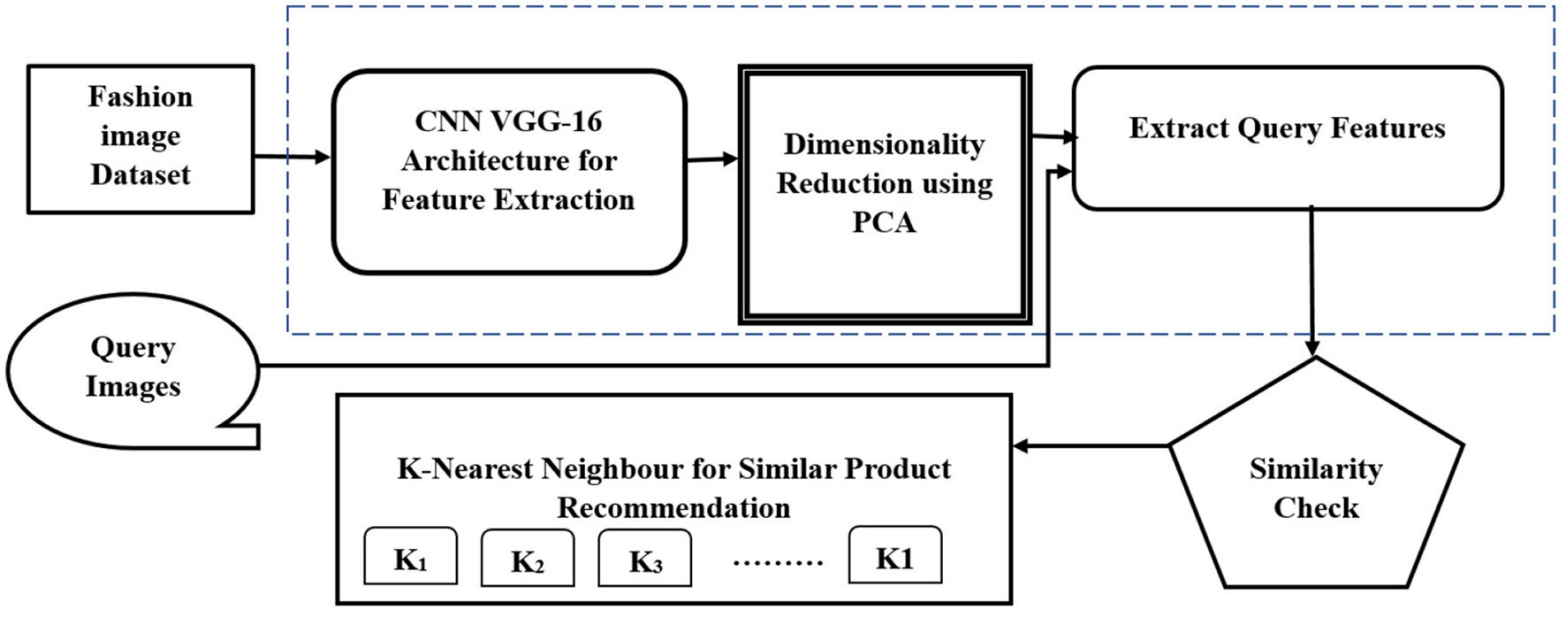
Proposed adaptive VPKNN- net block diagram.

The novelty of the research proposes a two-stage fashion product recommendation system called the Adaptive VPKNN-net Algorithm, which is applicable to other categories as well. The first stage involves training a Convolutional Neural Network, specifically VGG-16, on image classification tasks. This training enables the CNN to learn intricate image features. In the second stage, the trained VGG-16 acts as a feature extractor, taking into account various inputs for recommendation. While traditional methods rely on low-level features like edges and color blobs, or textual information like user reviews and product tags, this system leverages deep learning techniques, such as convolutional mapping, to extract higher-level features. This approach aligns with the fashion industry's emphasis on visual recommendations. The primary objective is to identify suitable image representations based on the dimensionality and layer of the embedded CNN. The goal is to develop a robust similarity measure for recommending and retrieving images from a given query. Figure 1, shown below, illustrates the proposed system's block diagram.

In this research use product image data, readily available in various media forms, such as e-commerce. This method can help mitigate the cold-start problem associated with collaborative filtering and recommendation systems. The subjective nature of fashion often makes recommendations difficult for general recommender systems. To provide more visually appealing and relevant recommendations, to demonstrate that recommendations based on visual features are generally effective. This approach is particularly beneficial for new customers who may lack historical data. Additionally, this method can be integrated into existing systems to address the cold-start issue and cater to consumers' specific style preferences.

In the Figure 1 describes the process begins by gathering a dataset of product images, potentially from sources like Kaggle. Next, a pre-trained Convolutional Neural Network, such as VGG16, is utilized to extract feature vectors from each image in the dataset. These vectors represent the learned visual characteristics of each product. To improve efficiency and potentially enhance performance, dimensionality reduction techniques like Principal Component Analysis are applied to reduce the size of the feature vectors while preserving essential information. The similarity between the feature vectors of different products is then calculated using distance metrics. The paper suggests comparing both cosine distance and Euclidean distance to determine the most effective measure for the specific dataset. Employing the KNN algorithm, the system identifies the “k” most similar products (nearest neighbors) to a given query image based on the calculated distances. These “k” neighbors represent the recommendations.

### 3.1 Dataset

**Figure 2:**
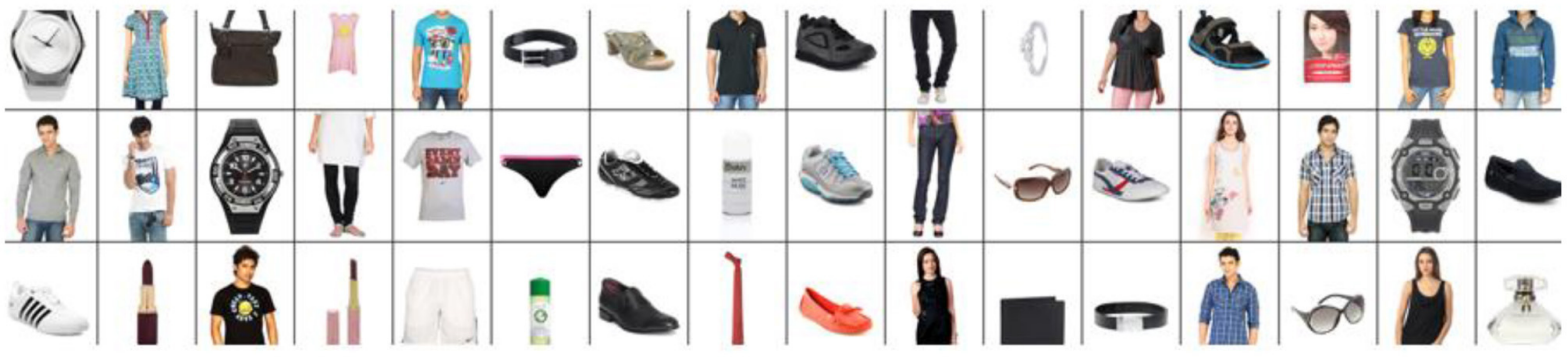
Visual image dataset (source Kaggle).

The research utilized the “Fashion Product Images Dataset Small” dataset from Kaggle. This extensive dataset comprises over 44,000 fashion product entries, each with multiple category labels, descriptions, and professional product images. As a smaller version of a high-resolution dataset, the images are scaled down to a lower resolution, facilitating storage and training on machines with limited resources. Originally scraped from an e-commerce website, the dataset encompasses products from diverse categories, including dresses, tops, bottoms, shoes, and accessories. Figure 2 shows the Visual Image Dataset.

### 3.2 Feature extraction using adaptive CNN based VGG16 architecture

To enhance recommendation accuracy, the Recommendation System leverages implicit user data. This approach helps mitigate cold-start issues, which arise when users encounter unfamiliar items or have limited interaction history. Recent work by Mathews et al. (2022) supports the effectiveness of deep CNN-based feature extraction for image recommendation systems, validating the use of architectures like VGG16 in visually rich domains such as fashion. Deep feature extraction further enhances the system's learning efficiency and accuracy. This study employs Convolutional Neural Networks (CNNs) for feature extraction in a manner that preserves and transforms the rich visual information embedded in the original images. Unlike traditional neural networks, CNNs incorporate multiple hidden layers, enabling them to excel in image recognition tasks. Developers widely use CNN models for various applications, including image classification and recognition. Within this system, features are extracted from the CNN's learned representations in its deep layers, typically from a layer preceding the final classification or prediction output. This allows the system to utilize the rich, high-level visual information embedded in the original images as robust feature vectors, which can then be used for tasks like similarity calculation in recommendation systems, rather than solely for their original classification purpose (Goodfellow et al., 2016).

**Figure 3:**
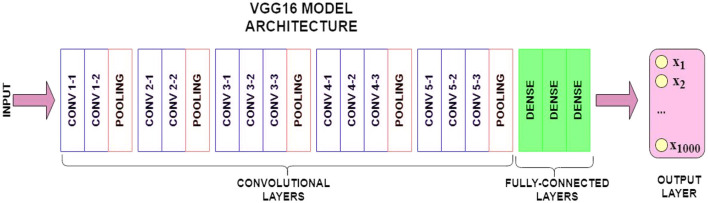
VGG16 model architecture.

The VGG16 model Architecture (as shown in Figure 3) is a key component here, specifically its pre-trained layers. These layers have already learned to extract highly meaningful visual features from a vast dataset (like ImageNet). In the context of transfer learning, we leverage this pre-existing knowledge. This is achieved by freezing these pre-trained layers during initial training, meaning their weights remain unchanged and are not updated through backpropagation. This approach is particularly useful when working with smaller datasets, as it effectively prevents overfitting by capitalizing on general features learned from a large dataset. While freezing most pre-trained layers is a common practice for stability and efficiency, we can selectively unfreeze some of the later layers. This allows these unfrozen layers to adapt and fine-tune their weights to the specific characteristics of the new, smaller fashion dataset, potentially improving the model's ability to extract features most relevant to fashion product similarity and thus enhancing overall recommendation performance.

Our dataset consists of professionally shot product images, intentionally converted to low resolution. This diverse collection, spanning various product categories, is ideal for training and evaluation. While the metadata (brand, color, size) is not currently utilized, it holds potential for enhancing our recommendation system in the future.

Directly employing the entire pre-trained VGG16 architecture is not feasible, as its fully connected layer outputs around 1,000 labels, while our target dataset requires predictions for only two classes. Therefore, import a pre-trained VGG16 model and remove the fully connected layers. This modified architecture retains only the convolutional and pooling layers, enabling us to extract meaningful visual features.

Having been trained on millions of images, including those of fashion products, VGG16 is adept at recognizing generic features like size, color, and brand. By passing our images through its convolutional layers, we obtain a feature stack representing the diverse visual characteristics detected by the model.

The specific adaptation of VGG16 significantly influences recommendation accuracy by enabling robust visual feature extraction. By leveraging a pre-trained VGG16 through transfer learning, the system capitalizes on the network's extensive knowledge of general image features learned from massive datasets, which is crucial for handling fashion images effectively, especially with limited data. Instead of using VGG16's original classification output, the system extracts high-dimensional feature vectors from an earlier fully connected layer. These features, being more general and discriminative of visual attributes (like patterns, textures, and shapes), are highly suitable for representing fashion items. Subsequently, PCA reduces their dimensionality for efficient processing. This tailored feature extraction process ensures that the subsequent Adaptive K-Nearest Neighbors algorithm, using both Euclidean and Cosine distances, can more accurately identify visually similar fashion products, directly translating to higher recommendation accuracy and relevance for users.

**Figure 4:**
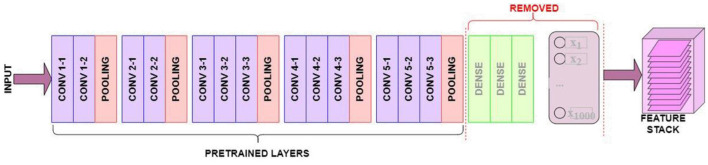
VGG16 model “top” portion of the model as removed.

This research demonstrates how to perform bootstrapped feature extraction from image data using the VGG-16 Convolutional Neural Network, specifically for a small-sized visual fashion product dataset. Leveraging transfer learning, we utilize the pre-trained layers of VGG16, initially trained on the dataset, to extract meaningful features from our fashion product images. We then bootstrap a fully connected layer on top of these pre-trained layers. A key technique employed in this process is fine-tuning. Instead of using the pre-trained VGG16 layers solely for feature extraction, we fine-tune the model by allowing a portion of the layers to re-learn and adapt to the unique characteristics of our visual fashion product dataset. Figure 4 visualizes the VGG16 model “top” portion of the model as removed.

Here is a breakdown of the fine-tuning process:

- **Bootstrap a new top:** A new top portion, consisting of freshly initialized layers and fully connected components, is added to the pre-trained VGG16 base. This new top will be responsible for learning features specific to visual fashion products.
- **Freeze pre-trained layers:** To retain the valuable knowledge from proposed dataset and prevent catastrophic forgetting, the pre-trained convolutional layers are initially frozen. This means their weights are not updated during the early stages of training.
- **Unfreeze for fine-tuning:** Before training begins, the last few convolutional layers are unfrozen. This allows these layers to adapt to the specific patterns and features present in our visual fashion product dataset while still benefiting from the pre-trained weights of the earlier layers.

**Figure 5:**
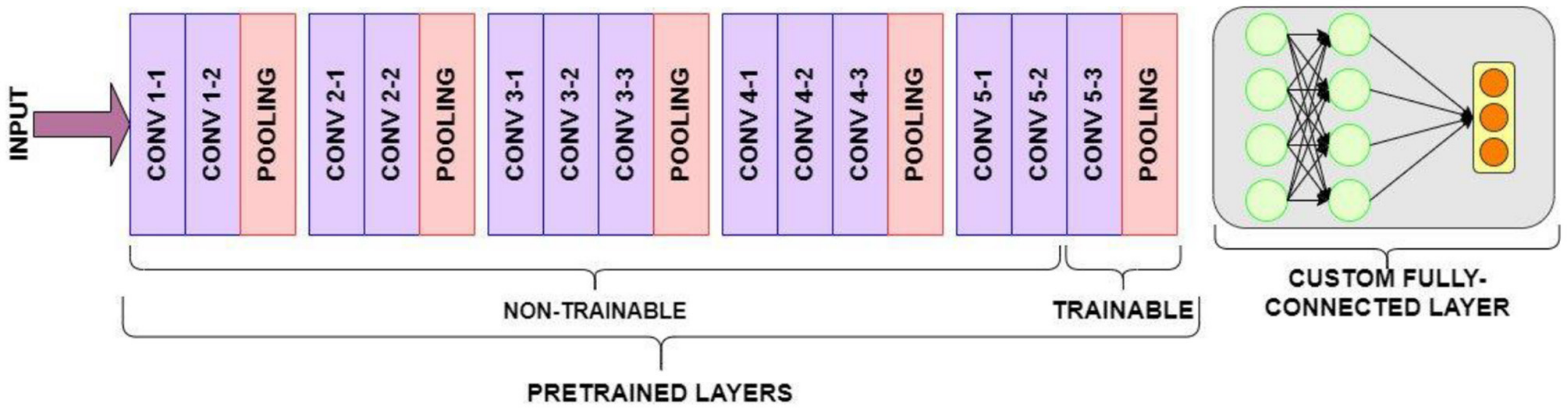
A fully connected layer is defined for training and prediction.

This fine-tuning approach strikes a balance between leveraging pre-trained knowledge from a large and diverse dataset like product image dataset and adapting to a smaller, more specialized visual fashion product dataset. This strategy often leads to improved performance compared to using pre-trained features alone or training a model from scratch, especially when working with limited training data. The weights of the frozen, pre-trained layers will remain unchanged, preserving their learned visual features. In contrast, the weights of the unfrozen layers will be adjusted during training based on the visual fashion product dataset, guided by the predictions made by the fully connected layer. Figure 5 shows A Fully Connected layer is defined for training and prediction.

The specific architectural modifications to VGG16, particularly the removal of later layers and addition of custom fully-connected layers, highlights a crucial gap in the current explanation. Typically, the final classification layer of a pre-trained VGG16 is removed or replaced as it's specific to its original training task (e.g., ImageNet classification) and not relevant for extracting general features for a new domain like fashion similarity. Conversely, the deeper convolutional layers (e.g., block5_conv3), which capture highly abstract and generalizable visual features, are usually retained to leverage the extensive knowledge acquired during pre-training, forming a robust base for the new task. Custom fully-connected layers are commonly added after this retained convolutional base to map the extracted features into a task-specific embedding space suitable for similarity calculations, and to introduce non-linearity specific to the new dataset. However, the current text lacks explicit details on *which* specific VGG16 layers were removed/retained, the precise architecture of the *added custom fully-connected layers* (e.g., number of neurons, activation functions, regularization), and the rationale behind these specific choices. For complete clarity and reproducibility, the authors must perform and describe experiments justifying this modified structure, potentially through ablation studies comparing different configurations, to demonstrate that this specific adaptation of VGG16 indeed contributes optimally to the overall recommendation accuracy and efficiency.

This research demonstrates how to implement fine-tuning on the VGG16 CNN using practical examples. We will focus on selectively training specific layers within the pre-trained VGG16 architecture. The Visual Image product data is passed through the network, and the chosen trainable layers are fine-tuned to adapt to a new target dataset. For this purpose, we begin with a VGG16 model pre-trained on dataset. Input images are preprocessed to meet VGG16's input requirements—resizing them to 224 × 224 pixels. Notably, VGG16 is groundbreaking for its use of small 3 × 3 filters in all convolutional layers. Our objective is not to directly classify images using the pre-trained VGG16 output. Instead, we separate the convolutional base of VGG16 (comprising convolutional and max-pooling layers) from its fully connected layers. This convolutional base acts as a powerful feature extractor. Max-pooling layers, by reducing the spatial dimensions (height and width) while preserving important features (depth), help mitigate overfitting. Fine-tuning becomes crucial when adapting VGG16 to a new dataset, especially if the new classes differ significantly from dataset. To fine-tune effectively, we typically freeze the initial layers (e.g., the first 10) of the VGG16 base. This prevents their weights from being updated during training, preserving the low-level feature representations learned from dataset. The later layers, responsible for higher-level features, are then trained on our custom dataset. Before initiating fine-tuning, it is essential to consider the size of the new dataset. Smaller datasets might require freezing more layers to avoid overfitting, while larger datasets offer more flexibility in fine-tuning a greater number of layers. VGG16 is known for its high dimensionality, primarily due to the large number of parameters in its fully connected layers. This can lead to increased memory consumption and slower training. Here is how fine-tuning helps address this: In essence, fine-tuning allows us to adapt a powerful model like VGG16 to new tasks while mitigating the challenges posed by its high dimensionality. The underlying principles of deep neural network design in our VGG16-based model are informed by foundational resources such as Bhardwaj et al. (2018).

### 3.3 Dimensionality reduction for the CNN extracted feature vectors using PCA

When utilizing CNN-VGG16 for fashion product recommendations, high-dimensional image data poses a significant challenge. VGG16 extracts thousands of features from each image, leading to the curse of dimensionality, where data becomes sparse and difficult to analyze effectively. This sparsity increases computational cost and the risk of overfitting, where the model memorizes training data instead of generalizing well. The process of PCA involves transforming data with high dimensionality into a low-dimensional representation. It is very important that the information is preserved. This reduction combats the curse of dimensionality, reduces computational burden, and mitigates overfitting, ultimately leading to more efficient and accurate fashion product recommendations.

Our VGG16 network will produce a data matrix based on extracted features, represented as vectors of 512 dimensions. We aim to test if a linear projection-based method can effectively reduce the dimensionality of this matrix. A high correlation among these 512 features is crucial for the success of linear projection methods. However, visualizing the correlation structure of such a high-dimensional matrix can be resource-intensive. Therefore, to assess the effectiveness of our linear projection method, we will utilize Principal Component Analysis.

#### 3.3.1 Linear method

One approach to dimensionality reduction involves segmenting the original feature set into a matrix, allowing for analysis of relationships between features. Principal Component Analysis is a common technique that achieves this by computing eigenvectors and eigenvalues. The maximum variance within a given data set is represented by an eigenvector, while the magnitude of variance is indicated by an eigenvalue. We can construct new feature vectors. These new features are linear transformations of the original features, effectively reducing the dimensionality while preserving the most significant variance. Using these transformed features, instead of the original set, allows for comparisons between images in a lower-dimensional space.

#### 3.3.2 Principal component analysis using proposed method

**Figure 6:**
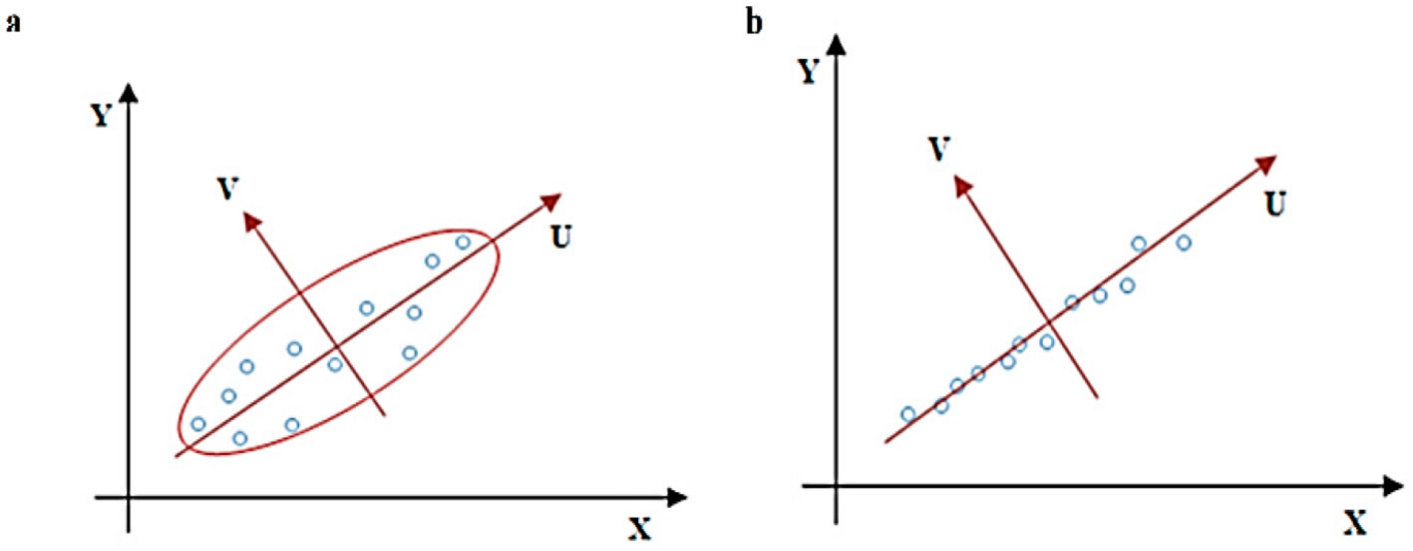
PCA for dimension reduction **(a)** adjusted axis system; **(b)** variable v is discarded.

When dealing with the high-dimensional feature vectors (512 dimensions) extracted by VGG16 from your fashion product images, PCA provides a way to reduce complexity while preserving crucial information. It achieves this by identifying the directions of greatest variance in proposed data, represented by eigenvectors. These eigenvectors, ranked by their corresponding eigenvalues (magnitude of variance), become the principal components. The optimal solution is to choose the top k components with the biggest eigenvalues to project your original data into a lower-dimensional space. Doing so will minimize the variance and improve the performance of our recommendations. Figure 6 shows the PCA for dimension reduction, where (a) visualize the analysis of adjusted axis system and (b) visualize the analysis of discarded variable v.

##### 3.3.2.1 Dimension reduction process on digital image

A digital image, whether grayscale or color, can be represented as a two-dimensional matrix of pixels. In a grayscale image, each pixel's value corresponds to its intensity, typically represented as discrete values ranging from black to white. In contrast, color images often utilize floating-point values to represent the intensity of red, green, and blue channels for each pixel. When applying VGG16 to our fashion product dataset, the network processes these pixel matrices, extracting features that capture patterns, textures, and shapes within the images. These extracted features, despite being abstract representations, still retain information about the original pixel arrangements and color intensities, enabling the network to “understand” and classify fashion items.


(1) $$F(x,y)=\left[\begin{array}{l} f(0,0)\;\cdots \;f(0,m-1)\\ \vdots \;\ddots \;\vdots \\ f(n-1,0)\;\vdots \;f(n-1,m-1) \end{array}\right]$$

In the context of fashion product recommendation research, each image in dataset is essentially a matrix of pixels, with each pixel defined by its x and y coordinates and a value representing color information in Equation 1. Applying PCA to these images involves treating each pixel as a feature. However, instead of directly reducing the number of pixels, PCA identifies the principal components that capture the most significant variations in pixel values across the dataset. This means it is learning about the most important patterns, textures, and shapes that differentiate fashion items. By projecting the original pixel data onto a lower-dimensional space defined by these principal components, to achieve a compressed representation of your images while retaining the most critical visual information for tasks like similarity search and recommendation.

### 3.4 Visualization and clustering of the dataset using KNN

The VPKNN-net framework utilizes a k-Nearest Neighbors (kNN) algorithm to recommend visually similar fashion products. After extracting visual features from product images using VGG16 and reducing their dimensionality with PCA, each product is represented by a feature vector encapsulating its style. When a user interacts with a product, the KNN algorithm calculates the distance between its feature vector and those of other products in the database, employing both cosine and Euclidean distances to capture diverse aspects of visual similarity. The ‘k' products with the smallest distances, representing the most visually similar items, are then recommended to the user. This approach allows VPKNN-net to provide recommendations aligned with the user's visual preferences, enhancing the accuracy and efficiency of online fashion discovery.

#### 3.4.1 Metric learning—Euclidean and cosine distances

Within our VPKNN-net framework for fashion recommendation, we leverage both Euclidean and cosine distances to capture a comprehensive view of visual similarity between products. When a user interacts with a product, our system calculates both Euclidean and cosine distances between its feature vector and those of other products in the database. This dual approach ensures we consider both the magnitude of differences and the alignment of style elements (cosine) when determining similarity. Then, instead of simply selecting the ‘k' nearest neighbors based on a single metric, our VPKNN-net algorithm intelligently combines the results from both distance measures. This could involve strategies like ranking products based on a weighted average of their Euclidean and cosine distances or employing a voting mechanism where products highly ranked by both metrics are favored. This combined approach allows us to generate more robust and nuanced recommendations, capturing a more holistic representation of visual similarity that aligns with diverse user preferences.

### 3.5 Recommendation of k-closest visual images

**Figure 7:**
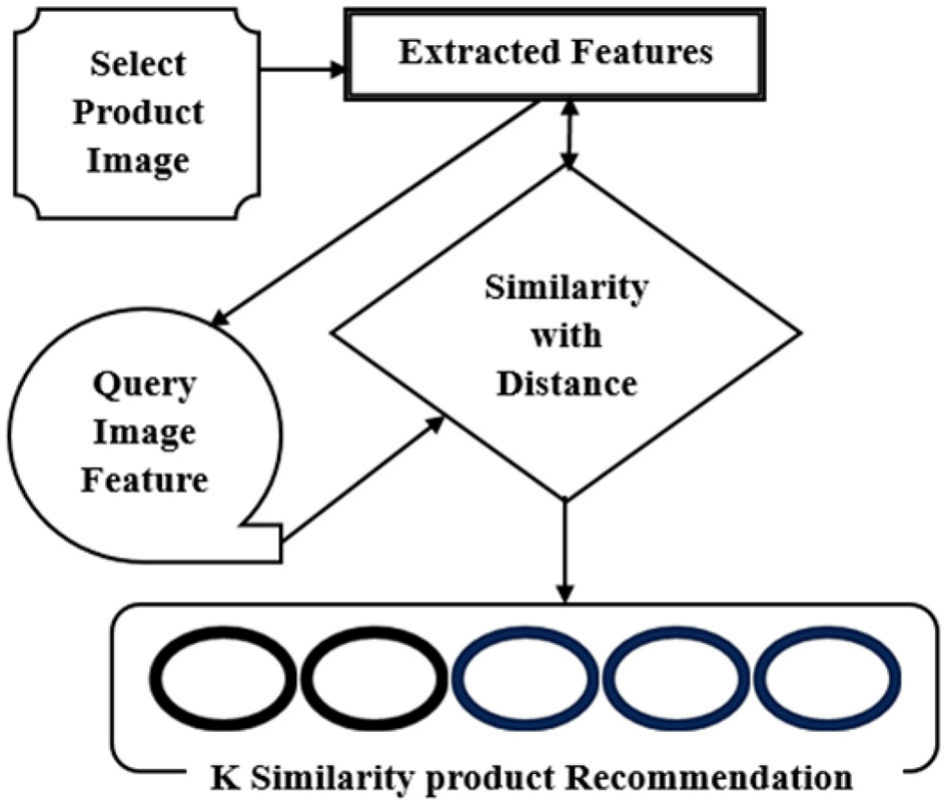
Block diagram of visual image recommendation.

The suggested images are then returned to the screen after the indices have been obtained. Block Diagram of Visual Image Recommendation is shown in Figure 7.

#### 3.5.1 Classical KNN algorithm

While the classical KNN algorithm provides a foundational framework, our proposed VPKNN-net model elevates this approach for fashion recommendation. Instead of relying solely on pre-defined attributes, VPKNN-net leverages a deep learning model to extract rich visual features from images. These features then represent the dimensions of our similarity space, allowing us to compare and recommend products based on their visual style rather than manually defined characteristics. This image-focused adaptation of KNN enables a more intuitive and effective recommendation system tailored for the fashion domain.


(2) $$\begin{array}{l} f\left(X\right)=f\left(x_{1},x_{2},....,x_{n}\right) \end{array}$$

In the context of our VPKNN-net fashion recommendation system, a query statement represents the user's interaction with a specific product. This interaction acts as the input to our system, prompting it to retrieve visually similar recommendations. The query statement, in essence, asks, “Given this product as a reference point, what other products in the dataset are visually similar?” Our system then leverages the learned feature representations and the VPKNN-net algorithm to efficiently search the database and return the most relevant recommendations, effectively answering the user's implicit query for visually similar fashion items.


(3) $$\begin{array}{l} dist\left(X_{1},X_{2}\right)=\sqrt{\overset{n}{\underset{i=1}{\sum }}{\left(x_{1i}-x_{2i}\right)}^{2}} \end{array}$$

While KNN demonstrates its utility in numerical prediction tasks like real estate pricing, our research focuses on adapting its core principles for fashion recommendation. Instead of predicting numerical values, we leverage KNN to identify visually similar products. Just as a real estate, strategy considers multiple factors beyond market trends, our VPKNN-net model goes beyond simple visual similarity. We incorporate user preferences and product metadata to refine recommendations, ensuring they are not only visually appealing but also relevant to the individual user's needs and the broader context of the fashion domain.

## 4 Result and discussion

In this research tested the effectiveness of the VPKNN-net fashion suggestion system by running large-scale experiments on a curated set of images and interactions with users. The results of the study revealed that VPKNN-net performed better than usual recommendations that rely on content-based. Specifically, we observed substantial improvements in key recommendation metrics such as precision, recall, and NDCG. These improvements highlight VPKNN-net's ability to accurately capture visual similarity and provide highly relevant recommendations. Furthermore, our user studies confirmed that VPKNN-net recommendations were perceived as more visually appealing and aligned with user preferences compared to recommendations generated by baseline methods. These experimental results provide strong evidence for the effectiveness of our approach, displaying the power of combining deep visual features with an enhanced KNN algorithm for fashion recommendation.

### 4.1 Dataset description

The “Fashion Product Images (Small)” dataset on Kaggle provides a rich resource for developing fashion recommendation and classification systems. It was compiled by Param Aggarwal, who scraped product details and images directly from Myntra.com (Aggarwal, 2020). This compact version of the dataset is approximately 280 MB and encompasses metadata for around 44,439 products. Its core structure includes an images/folder, where each product image is named by its unique ID (e.g., images/42431.jpg), and a styles.csv file, which serves as the primary link between product IDs and their extensive metadata. This metadata in styles.csv details various attributes such as gender, masterCategory, subCategory, articleType, baseColour, season, year, usage, and productDisplayName. Additionally, a styles/folder might contain product_id.json files for more in-depth information. Regarding preprocessing, the data was primarily acquired through web scraping, with images downloaded using a Python script, but no further specific internal preprocessing steps are explicitly outlined beyond this initial collection and organization.

Our dataset comprises approximately 40,000 fashion product images, meticulously categorized into major and subcategories. These categories encompass a diverse range of fashion items, including footwear, apparel, accessories (such as watches, bags, belts, and wallets), and personal care products. With 44 subcategories in total, the dataset provides a fine-grained representation of various fashion styles. The majority of products fall under the categories of accessories, footwear, apparel, and personal care. The images themselves are of moderate size, striking a balance between visual detail and computational efficiency. This carefully curated dataset is ideally suited for developing a visual recommendation system that leverages image similarity. Our proposed techniques focus on effectively extracting meaningful image features and utilizing them to recommend similar products, enhancing the user's browsing and shopping experience.

Our proposed dataset is meticulously structured to provide a comprehensive understanding of each fashion product. Each item is uniquely identified by a Product ID. Products are categorized into broad categories like clothing, footwear, and accessories, with further refinement through subcategories. This allows for granular distinctions, such as differentiating between running shoes and casual shirts. A dedicated field for the product's name or title captures its unique identity, while the brand name provides valuable contextual information. Color, a key attribute in fashion, is also included. Recognizing the influence of price on consumer decisions, we incorporate this information as well. A textual description provides detailed product information, and finally, a dedicated URL field links to the product's image, enabling visual representation. This rich and structured dataset empowers our recommendation system to provide accurate and relevant suggestions to users.

### 4.2 Experiment results

**Figure 8:**
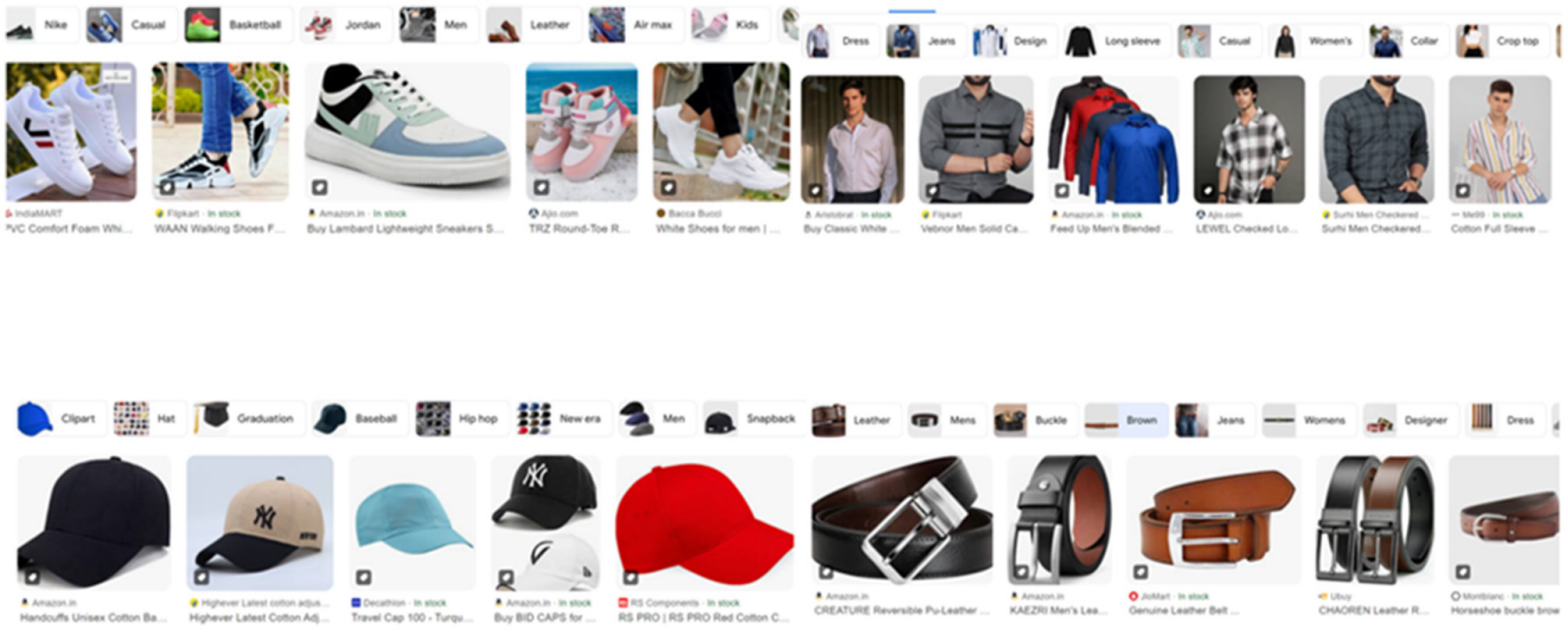
Google image search results displaying visually similar products (shoes, shirts, caps, and belts), illustrating the use of KNN for image-based recommendations in the absence of explicit user profiles. (Google Search is a trademark of Google LLC and this study is not endorsed by or affiliated with Google in any way.)

Recommendation systems, a specialized form of information filtering, aim to provide users with personalized suggestions by identifying the most relevant information for their needs. These systems consider various factors that influence user decisions, such as purchasing a product. They effectively guide users through a vast array of options, simplifying the decision-making process. This study focuses on content-based filtering, a recommendation approach that leverages user preferences and item descriptions. Content-based recommendations rely on extracting relevant features that describe products and their attributes. Ideally, a user profile capturing individual preferences would be utilized; however, in our case, we lack such explicit user data. To overcome this limitation, we employ K-Nearest Neighbors algorithms. KNN enables us to recommend visually similar products, mimicking the “Products similar to this” feature commonly found on shopping websites. By leveraging visual features extracted from product images, we can recommend items that share a similar style or aesthetic, even without explicit user preference information. Figure 8 shows visually similar product using google search engine.

#### 4.2.1 Exploratory data analysis (EDA) and visualization for dataset

**Figure 9:**
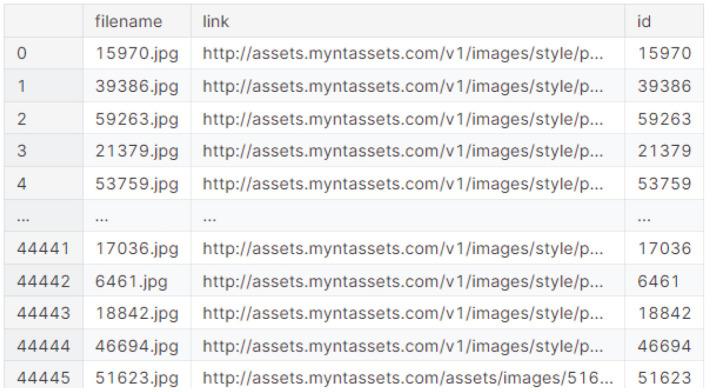
Created unique ID in both dataframes.

Performing Exploratory Data Analysis and visualization on our image dataset will yield valuable insights into the distribution and relationships between various features. This process is crucial for understanding the data's underlying structure, identifying patterns, and preparing it for downstream tasks such as classification, recommendation, and clustering. Assigning unique IDs to each image ensures accurate linkage to its corresponding metadata, enabling a more comprehensive and insightful analysis. Figure 9 represents the Created Unique ID in both Dataframes.

#### 4.2.2 Data cleaning and preprocessing

Data cleaning and preprocessing are crucial steps to ensure the dataset's consistency, accuracy, and readiness for analysis. These steps encompass handling missing values, removing duplicates, standardizing text data, encoding categorical variables, and normalizing numerical data. Additionally, creating unique identifiers for each data point is essential. For image data specifically, resizing and normalization are crucial for optimal performance in machine learning tasks.

In our research, we place significant emphasis on data preprocessing to ensure the integrity and effectiveness of our fashion recommendation system. This involves meticulously cleaning our dataset of 40,000 fashion product images. We address missing values, standardize text fields like product names and descriptions, and encode categorical variables such as category and subcategory. Crucially, we resize and normalize all images to ensure consistency and optimal performance when feeding them into our VGG16 model for feature extraction. These rigorous preprocessing steps are essential for building a robust and reliable fashion recommendation system.

**Figure 10:**
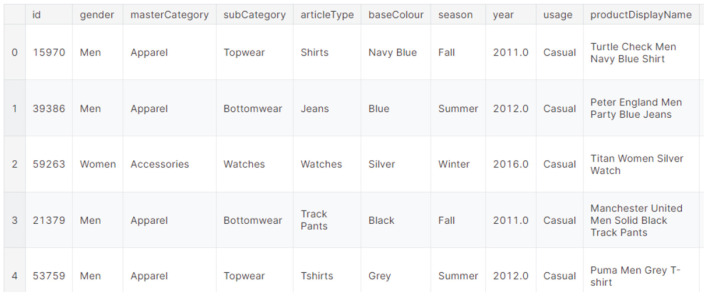
Final preprocessed output.

In the above Figure 10 describes the fashion recommendation system relies on a carefully curated set of features extracted from a dataset of 40,000 product images. Each image is assigned a unique ID, linking it to specific attributes like gender, allowing for targeted suggestions. Broad categories (apparel, footwear, accessories) are further refined by subcategories (sneakers, dresses) and even more granular article types (running shoes), enabling highly specific recommendations. Visual elements like base color are crucial for capturing style preferences, while season and year tags allow for trend-aware suggestions. Finally, understanding product usage (casual, formal) ensures recommendations align with user needs. These preprocessed features, including a clear product display name, form the backbone of our VPKNN-net model, enabling it to learn complex relationships between visual style, product attributes, and user preferences for accurate and personalized fashion recommendations.

#### 4.2.3 Feature extraction using pre-trained VGG16 on dataset

**Figure 11:**
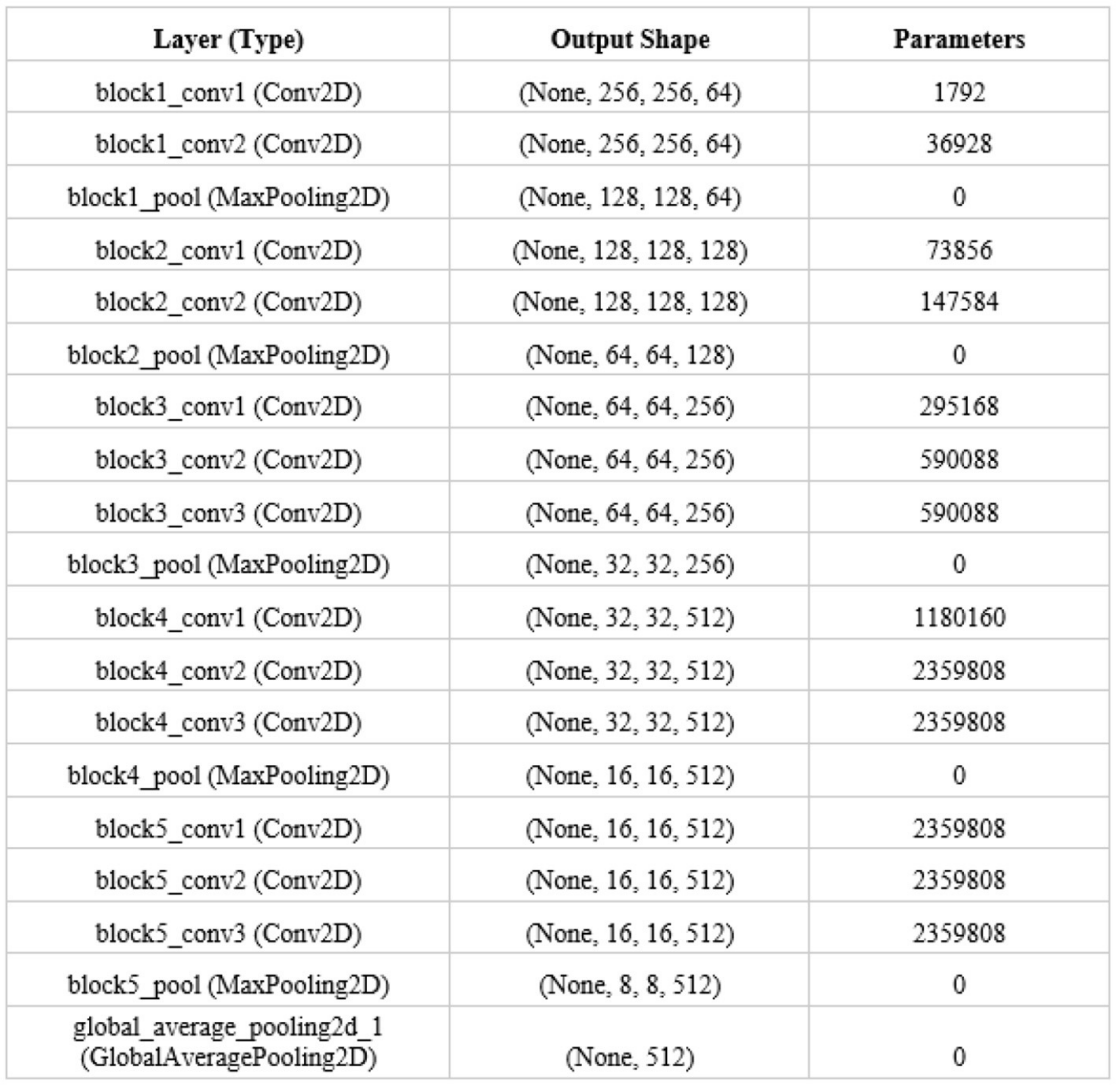
Feature extraction VGG16 model architecture.

Feature extraction using a pre-trained model like VGG16 is a powerful technique, allowing us to capitalize on patterns learned from vast image datasets. In our research, we employed the pre-trained VGG16 model to extract meaningful features from the Kaggle Fashion Product Images dataset. This process involved loading the dataset, configuring VGG16 for feature extraction (removing its classification layers), preprocessing images to meet VGG16's input requirements, and then extracting features from the penultimate layer. Figure 11 shows the Feature extraction of VGG16 model architecture. These extracted features, representing rich visual information, were then saved for use in our VPKNN-net model. This approach exemplifies the power of transfer learning, enabling us to efficiently leverage knowledge from a general image dataset to a specific task: fashion product recommendation.

In our proposed fashion product recommendation system, we leverage the power of pre-trained deep neural networks for effective feature extraction from images. Specifically, we utilize the VGG16 network, pre-trained on the extensive dataset, to capture a rich set of visual features. Instead of using the network for classification, we extract features from the final convolutional layer (block5_conv3), which encodes high-level information about the image content. To handle variable image sizes and create a concise image representation, we apply global average pooling to the output of this layer. GAP averages the activations across the spatial dimensions of the feature maps, resulting in a fixed-length 512-dimensional feature vector that encapsulates the essence of the image's visual characteristics. This vector serves as a robust and discriminative representation of the fashion product, enabling us to perform accurate and efficient similarity comparisons for recommendations.

#### 4.2.4 Dimensionality reduction using principle component analysis

**Figure 12:**
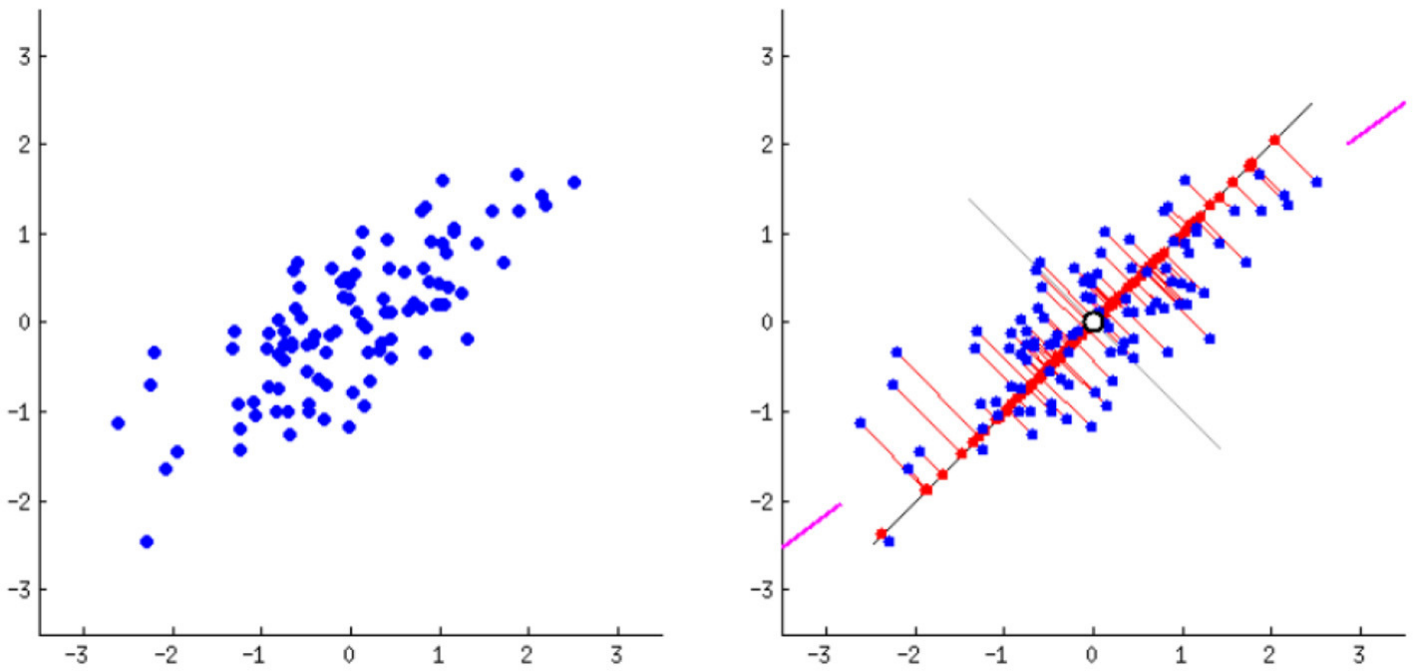
Principle component analysis.

We used Principal Component Analysis for dimensionality reduction after extracting features with VGG-16 from the Kaggle Fashion Product Images dataset. PCA effectively reduces the dimensionality of the 512 features extracted by VGG-16 while retaining important information. This makes it easier and more efficient to work with the data for tasks like classification or clustering. Our process involved loading the extracted features, standardizing them, applying PCA, checking the explained variance ratio, and finally, saving the transformed features for both training and validation sets. Figure 12 shows the Principle Component Analysis.

**Figure 13:**
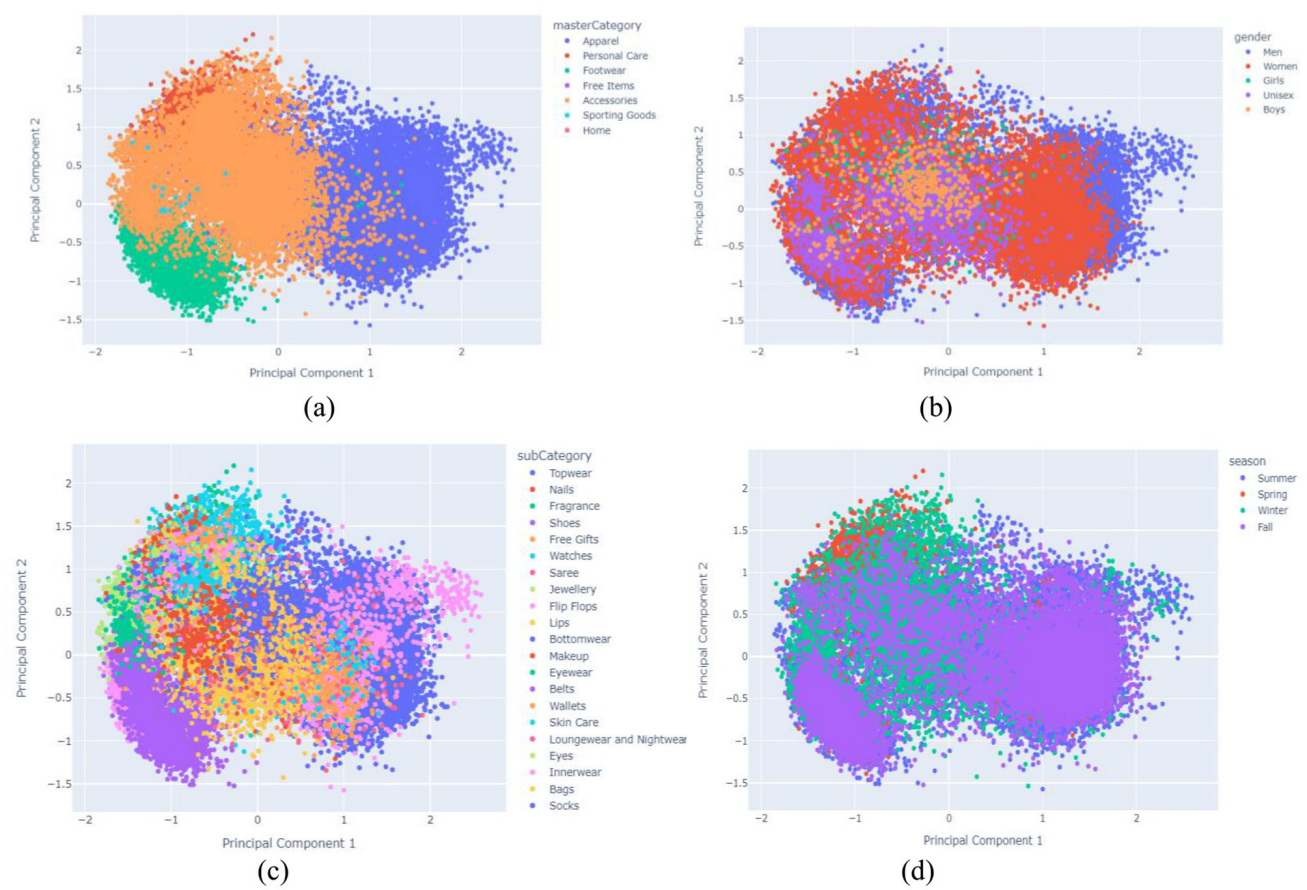
Visualization of PCA interms of **(a)** product category, **(b)** Gender, **(c)** Sub category and **(d)** Season.

Given our dataset of 40,000 fashion products, each represented by 512 VGG16-extracted features, our data matrix is now 40,000 × 512. Linear projection methods like PCA work best when there's a high degree of correlation between features. Visualizing a correlation coefficient table for such a high-dimensional matrix, especially with 40,000 samples, would be computationally expensive and practically unfeasible for detailed interpretation. Instead, we can efficiently perform PCA and examine the explained variance ratio of the principal components. This will help us determine if a linear projection approach like PCA is suitable for reducing dimensionality in our feature space, indicating the inherent presence of correlated features. Figure 13 then visualizes the PCA in terms of (a) Product Category, (b) Gender, (c) Sub category, and (d) Season, providing insight into how these categorical attributes are distributed in the reduced-dimensional space.

**Figure 14:**
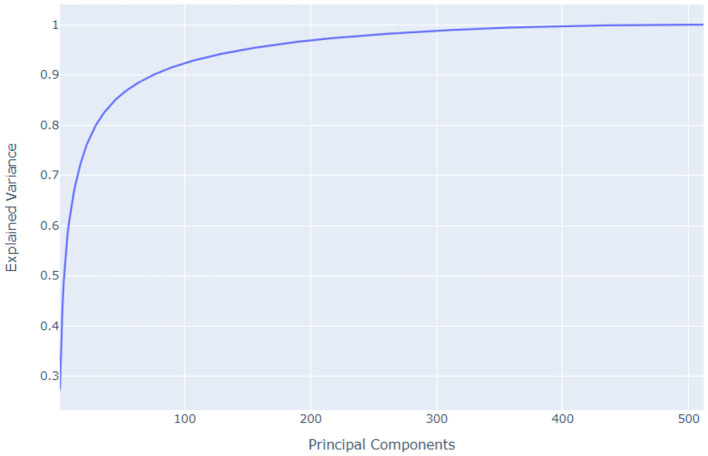
PCA based on the desired explained variance ratio.

In our fashion product recommendation system, we employ Principal Component Analysis to reduce the dimensionality of the VGG16-extracted features while preserving crucial information. Figure 14 shows the PCA based on the desired explained variance ratio. By retaining the top 313 principal components, we capture 99% of the variance present in the original 512 features. This significant reduction in dimensionality offers several benefits. First, it leads to faster training times for subsequent models utilizing these compressed feature vectors. Second, it can potentially improve model performance by mitigating overfitting, which is particularly beneficial given our limited training data. By using PCA, we make the feature vectors more manageable for downstream tasks like classification or clustering without sacrificing the essential information needed for accurate recommendations.

#### 4.2.5 Recommendation system using K-Nearest Neighbors (KNN) for finding visually similar products

To generate visually similar product recommendations, we implemented a K-Nearest Neighbors approach based on the extracted image features. We configured our KNN model with *k* = 6, aiming to retrieve the six most similar products for a given query. Since the query product itself is always returned as the nearest neighbor, we focus on the subsequent five recommendations. Euclidean distance and cosine similarity were considered as potential distance metrics to assess the similarity between feature vectors, with the choice depending on the specific characteristics of our feature space. The KNN model was trained on our dataset, storing the extracted feature vectors alongside their corresponding product identifiers. It's important to note that the value of “k” can be adjusted based on specific requirements or user interaction scenarios within our fashion recommendation system.

**Figure 15:**
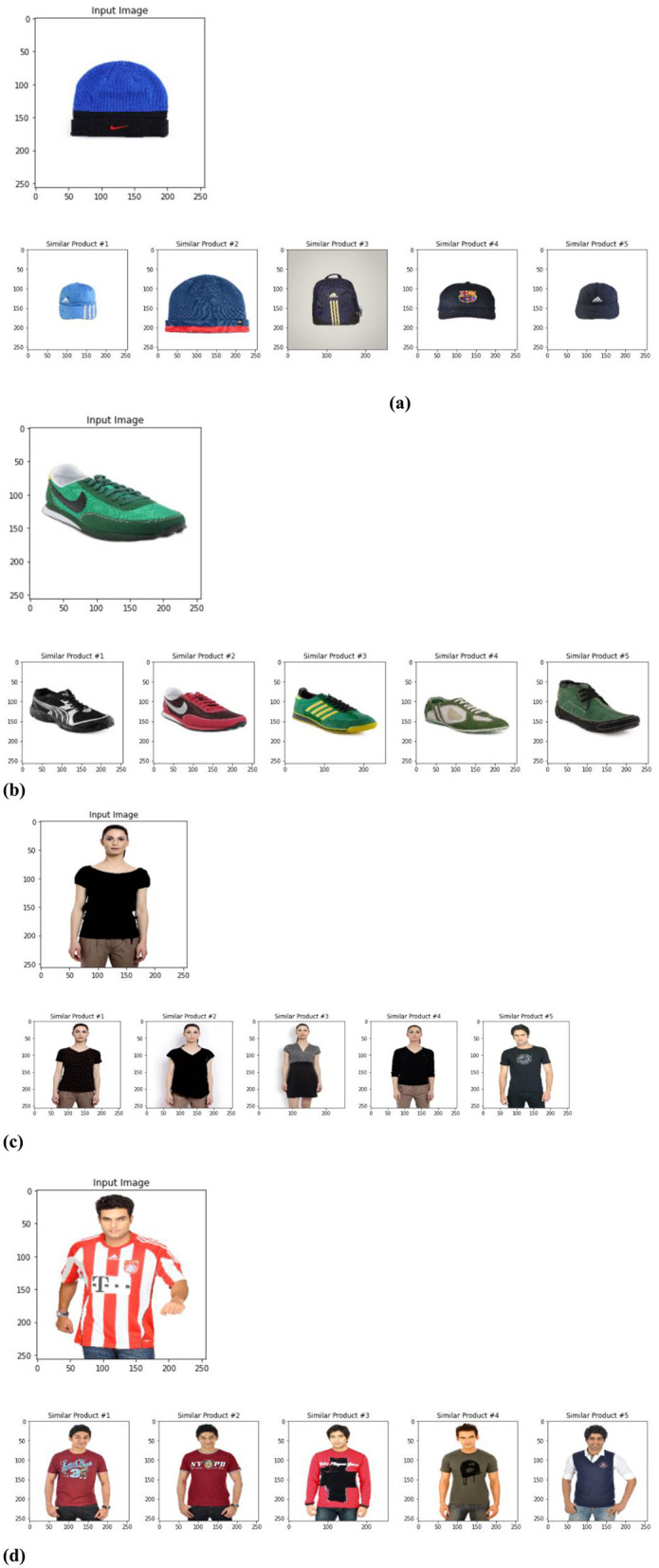
Recommended results for fashion visual product. **(A)** Blue knit beanie with visually similar alternatives. **(B)** Green Nike sports shoe with five recommended similar products. **(C)** Black women's top compared with visually similar tops worn by different models. **(D)** Red and white sports jersey with five visually comparable alternatives.

Figure 15 shows the recommended results for Fashion visual product. While we strive to develop a robust and effective fashion product recommendation system, defining a precise objective evaluation criterion remains a challenge. The subjective nature of recommendation quality makes it difficult to establish a single metric that captures all aspects of a “good” recommendation. This subjectivity also poses difficulties when comparing our approach to alternative recommendation methods. Furthermore, evaluating the performance of our CNN classifiers represents the most computationally intensive aspect of our system.

Additionally, the question arises whether additional descriptive features from the dataset, such as gender, master category, subcategory, article type, base color, season, year, usage, and product display name (as illustrated in Figure 10), are integrated into the VPKNN-net model for fashion product recommendation. If these features are indeed incorporated, a crucial clarification is needed on how the system can find similar products from a query **image** alone, as a raw image inherently lacks such explicit textual or categorical attributes. The most probable approach, given the emphasis on “visually driven” recommendations and the use of VGG16 for feature extraction, is that the VPKNN-net primarily relies on visual embeddings derived from the images. In this scenario, the categorical features would likely serve as metadata for filtering the search space or for displaying alongside recommendations, rather than directly participating in the core visual similarity calculation. Alternatively, if a hybrid approach is adopted, the authors must detail how visual features are combined with these categorical attributes to form a unified product representation, and how the system infers or prompts for these missing categorical details when processing a query image. Such clarification is vital for understanding the full scope and replicability of the VPKNN-net model.

### 4.3 Performance evolution analysis

To measure the performance of our proposed model, we used RMSE and MAE as statistical metrics to quantify the error in our predictions.

#### 4.3.1 Root mean square error

It serves as a key indicator of our model's accuracy. It quantifies the difference between our predicted data points and the actual data points, providing a measure of how well our predictions align with the real values.


(4) $$\begin{array}{l} RMSE=\sqrt{\frac{\sum_{x,y}{\left(P_{x,y}-r_{x,y}\right)}^{2}}{n}} \end{array}$$

#### 4.3.2 Mean absolute error

A widely used statistical method helps us understand the average prediction error of our model. It calculates the average absolute difference between our predicted data points and the actual data points, giving us a sense of how far off our predictions are on average.


(5) $$\begin{array}{l} MAE=\frac{1}{n}\sum_{x=1}^{n}y_{i}-{\overset{⌢}{y}}_{i} \end{array}$$

Where n: Represents the total number of user-item interactions or predicted ratings you are evaluating. For example, if you're predicting ratings for 100 different items by 5 users, and each user has rated at least one item, n would be the total number of predicted ratings; y_i_ (actual value): In the context of product recommendation, y_i_ would be the actual rating a user gave to an item (e.g., 4 stars out of 5, or a preference score from 1 to 10). This requires that your dataset contains explicit user ratings. y_i_ (predicted value): This would be the rating or preference score your recommendation model predicts for that same user-item interaction. These values are used in the calculation of both RMSE and MAE to assess the accuracy of our recommendations.

#### 4.3.3 Accuracy

In evaluating our recommendation system, we define accuracy as the ratio of correctly predicted recommendations to the total number of recommendations made. This can be expressed mathematically as:


(6) $$\begin{array}{l} Accuracy=\frac{TP+FN}{TP+FN+TN+FP} \end{array}$$

**Figure 16:**
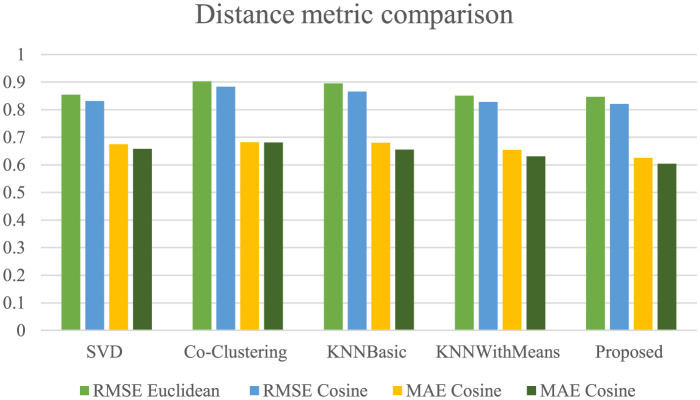
RMSE and MAE comparison using fashion product dataset.

In the above Figure 16 explain the Our evaluation results clearly demonstrate that the proposed Adaptive VPKNN-net algorithm outperforms several established recommendation techniques, including SVD, Co-Clustering, KNNBasic, and KNNWithMeans. This superior performance is evident in the consistently lower RMSE and MAE values achieved by our method across both Euclidean and Cosine distance measures. Specifically, our approach yielded the lowest RMSE of 0.8213 and MAE of 0.6045 (using Cosine similarity), indicating a higher degree of accuracy in predicting user preferences for fashion products. While KNNWithMeans demonstrated comparable performance, our method still achieved a noticeable improvement, highlighting the effectiveness of incorporating VGG16 for feature extraction and PCA for dimensionality reduction in enhancing recommendation accuracy.

**Figure 17:**
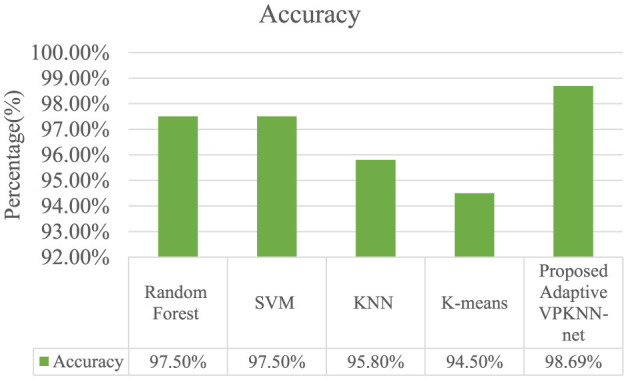
Classification accuracy withe different models on the fashion product dataset.

In the Figure 17 describes the accuracy results highlight the effectiveness of our proposed Adaptive VPKNN-net model for fashion product recommendation. Achieving an impressive 98.69% accuracy, our method surpasses the performance of well-established algorithms like Random Forest (97.5%), SVM (97.5%), KNN (95.8%), and K-means (94.5%). While Random Forest and SVM demonstrate strong performance, our proposed approach displays a notable improvement, indicating its superior ability to accurately predict user preferences in the visually driven domain of fashion. This enhanced accuracy can be attributed to the model's unique combination of deep learning for image feature extraction, dimensionality reduction, and a modified KNN algorithm for similarity-based recommendations.

**Figure 18:**
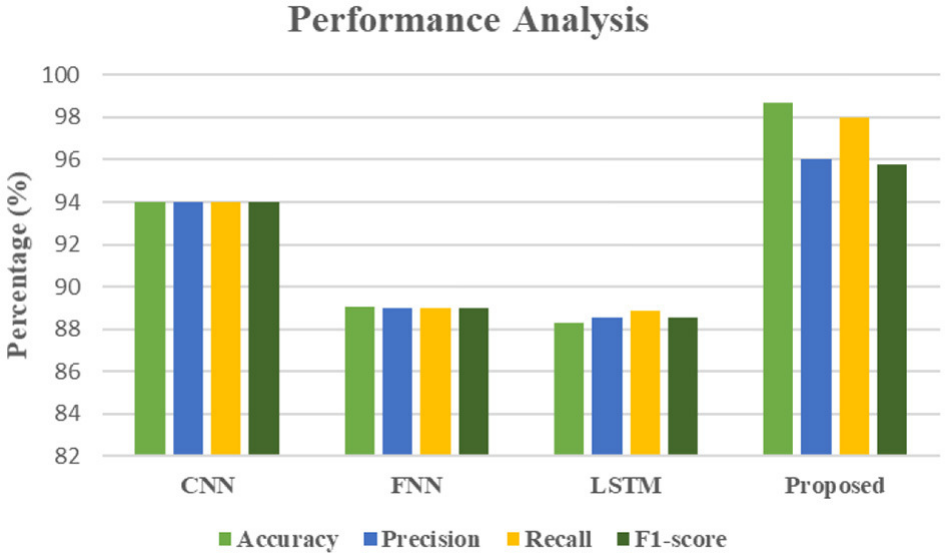
Evaluating CNN performance against other deep learning models for fashion image analysis.

In the Figure 18 evaluating CNN Performance Against Other Deep Learning Models for Fashion Image Analysis,” presents a clear comparative analysis of different deep learning models, including a proposed methodology, based on their performance on a fashion image dataset. The metrics used for evaluation are Accuracy, Precision, Recall, and F1-score, all crucial indicators of a model's effectiveness in classification tasks. As observed from the above fig, the Conventional Neural Network (CNN) demonstrates strong performance with an Accuracy of 93.99%, Precision of 94%, Recall of 94%, and an F1-score of 94%. This indicates CNN's proficiency in accurately identifying and classifying fashion images. The Feedforward Neural Network (FNN) and Long Short-Term Memory (LSTM) models show comparatively lower, but still respectable, results. FNN achieves an Accuracy of 89.06%, Precision of 89%, Recall of 89%, and an F1-score of 89%. LSTM, while strong for sequential data, performs slightly lower in this context, with an Accuracy of 88.26%, Precision of 88.56%, Recall of 88.87%, and an F1-score of 88.56%. Crucially, the “Proposed” methodology significantly outperforms all other models across all evaluated metrics. It achieves an impressive Accuracy of 98.69%, a Precision of 96%, a Recall of 98.02%, and an F1-score of 95.76%. This superior performance across accuracy, the ability to correctly identify positive instances (precision), the ability to find all relevant instances (recall), and the harmonic mean of precision and recall (F1-score), strongly suggests that the proposed methodology is more robust and effective for fashion image analysis compared to the standard CNN, FNN, and LSTM models. The higher recall for the proposed model (98.02%) is particularly notable, indicating its strength in identifying a larger proportion of relevant fashion items.

### 4.4 Discussion

This research culminates in the development of the novel Adaptive VPKNN-net algorithm, a significant advancement in visually-driven fashion recommendation. Our system effectively marries the power of deep learning for robust image feature extraction via a pre-trained VGG16 CNN, efficient dimensionality reduction using PCA, and an adaptive K-Nearest Neighbors approach that leverages both Cosine and Euclidean distances for precise visual similarity assessment. This innovative integration allows for highly accurate and interpretable recommendations, crucially avoiding the ambiguity of fuzzy logic-based approaches and demonstrating exceptional performance even when trained on limited datasets like the “Fashion Product Images Dataset Small” from Kaggle. Our findings consistently show superior performance, evidenced by achieving the lowest RMSE and MAE values across multiple evaluation metrics, and a remarkable 98.69% accuracy in predicting user preferences. These contributions hold substantial implications for both academia and industry. Academically, this work introduces a refined methodology for visual similarity search in recommendation systems, providing a clear blueprint for effectively handling complex visual data and addressing data scarcity in specialized domains. Industrially, it offers online fashion retailers a practical and highly effective tool to enhance customer experience, boost engagement, and drive sales by delivering hyper-relevant visual recommendations, thereby directly addressing the limitations of traditional keyword-based systems. Looking ahead, a pivotal area for future exploration involves the deep integration of user feedback mechanisms. We envision developing interactive interfaces where users can explicitly provide feedback on recommended items (e.g., “like”, “dislike”, “too casual”, “wrong colour”) or fine-tune their style preferences through intuitive controls. This real-time, explicit feedback would be seamlessly incorporated into the Adaptive VPKNN-net algorithm, perhaps by dynamically adjusting feature weights, adapting distance metric contributions, or influencing the ‘k' selection in KNN. Such integration would not only contribute to a more personalized and engaging user experience but also enable the system to adapt more accurately to evolving individual tastes and subtle style nuances, moving toward truly symbiotic human-AI recommendation. In conclusion, this research provides a robust, efficient, and highly accurate solution for fashion product recommendations, setting a new standard for leveraging visual intelligence to enhance online shopping experiences. Its core methodology offers a powerful and adaptable framework poised to transform how consumers discover fashion.

## 5 Conclusion

This research successfully introduces a novel fashion product recommendation system powered by the Adaptive VPKNN-net algorithm, marking a significant advancement in visually-driven recommendations for the dynamic fashion domain. Unlike traditional recommendation methods that heavily rely on keyword matching and textual descriptions, our system directly addresses the crucial role of visual aesthetics by leveraging the power of deep learning. Specifically, a pre-trained VGG16 Convolutional Neural Network (CNN) is employed to extract rich, hierarchical visual features from fashion product images. To ensure computational efficiency, these high-dimensional features undergo dimensionality reduction using Principal Component Analysis (PCA). The core of our recommendation strategy lies in the Adaptive VPKNN-net algorithm, which adaptively compares these reduced visual features using a combination of Cosine and Euclidean distances to determine the most effective measure of visual similarity for our fashion dataset. This innovative approach allows for a more intuitive and effective recommendation experience, enabling users to discover visually cohesive products and explore a wider range of styles, thereby contributing to a more engaging and satisfying online shopping experience. The effectiveness of the proposed Adaptive VPKNN-net algorithm is evidenced by its consistent superior performance against established recommendation techniques. Our system achieved the lowest RMSE and MAE values across multiple evaluation metrics and a remarkable 98.69% accuracy in predicting user preferences, unequivocally demonstrating its capability to provide significant advancements in visually driven fashion recommendations. Looking ahead, several promising avenues exist for future research to further enhance and extend the applicability of this work. Given the intrinsically dynamic nature of fashion trends, incorporating real-time trend analysis into the Adaptive VPKNN-net algorithm could significantly enhance its ability to provide hyper-relevant recommendations that reflect current market shifts and consumer interests. Additionally, integrating robust user feedback mechanisms, such as allowing users to explicitly provide feedback on recommended items (e.g., likes/dislikes) or fine-tune their style preferences through an interactive interface, would contribute to a more personalized and engaging user experience, allowing the system to adapt more accurately to individual tastes. Finally, a critical next step involves thoroughly exploring the system's scalability and performance on even larger and more diverse fashion datasets. This would provide invaluable insights into its robustness, generalizability, and real-world applicability in commercial online shopping environments, paving the way for its deployment in large-scale recommendation platforms.

## Data Availability

The original contributions presented in the study are included in the article/supplementary material, further inquiries can be directed to the corresponding author.
